# The Influence of Life History Milestones and Association Networks on Crop-Raiding Behavior in Male African Elephants

**DOI:** 10.1371/journal.pone.0031382

**Published:** 2012-02-08

**Authors:** Patrick I. Chiyo, Cynthia J. Moss, Susan C. Alberts

**Affiliations:** 1 Department of Biology, Duke University, Durham, North Carolina, United States of America; 2 Department of Biological Sciences, University of Notre Dame, Notre Dame, Indiana, United States of America; 3 Amboseli Trust for Elephants, Nairobi, Kenya; University of Western Ontario, Canada

## Abstract

Factors that influence learning and the spread of behavior in wild animal populations are important for understanding species responses to changing environments and for species conservation. In populations of wildlife species that come into conflict with humans by raiding cultivated crops, simple models of exposure of individual animals to crops do not entirely explain the prevalence of crop raiding behavior. We investigated the influence of life history milestones using age and association patterns on the probability of being a crop raider among wild free ranging male African elephants; we focused on males because female elephants are not known to raid crops in our study population. We examined several features of an elephant association network; network density, community structure and association based on age similarity since they are known to influence the spread of behaviors in a population. We found that older males were more likely to be raiders than younger males, that males were more likely to be raiders when their closest associates were also raiders, and that males were more likely to be raiders when their second closest associates were raiders older than them. The male association network had sparse associations, a tendency for individuals similar in age and raiding status to associate, and a strong community structure. However, raiders were randomly distributed between communities. These features of the elephant association network may limit the spread of raiding behavior and likely determine the prevalence of raiding behavior in elephant populations. Our results suggest that social learning has a major influence on the acquisition of raiding behavior in younger males whereas life history factors are important drivers of raiding behavior in older males. Further, both life-history and network patterns may influence the acquisition and spread of complex behaviors in animal populations and provide insight on managing human-wildlife conflict.

## Introduction

Factors that influence learning and the spread of behavior in wild populations of social animals are of interest in understanding the response of species to changing environments and for species conservation [1], [2]. These factors are relevant for understanding the prevalence of behaviors such as foraging on cultivated crops or livestock by wildlife. These foraging behaviors are a major cause of human-wildlife conflict and wildlife mortality in human dominated landscapes [3]–[5]. In species that raid cultivated crops, simple models of exposure of individual animals to crops do not entirely explain the prevalence of this behavior in populations of raiding species [6]–[8]. For example in the Amboseli elephant population, we estimated that 1/3 of all post-dispersal male elephants raid crops and apparently no females raid crops, but all males and most family groups have access to crops [9]. How individual elephants acquire crop-raiding behavior and why some elephants never adopt raiding even when they range in proximity to crops is not known. We explored the influence of life history factors and social network factors as drivers of crop raiding behavior in elephants. In this study we focused on males because we never detected raiding by females in our population [9]. In many elephant populations, males may be responsible for 70–100% of crop damage incidents in African elephants [10]–[13] and in Asian elephants [14], [15].

Life history milestones, being correlated with age, may influence crop raiding behavior in male elephants and possibly other large mammals in several ways. First, sexual maturation and attainment of a peak in reproduction represent life history milestones manifested by a rise in energy requirements [16]–[18]. Second, dispersal from a natal group or home range represents another life history milestone that may increase the probability that males encounter crops or become exposed to other raiding elephants. In elephants, sexual maturation, as indicated by age of first reproduction in males, occurs at 25–30 years and males attain their reproductive peak at 45–50 years [19]–[21]. For males that have attained reproductive age, reproductive success is positively correlated with musth duration and nutritional state [19], [20], [22], suggesting that intense physiological and nutritional demands are associated with reproduction. Such demands can provide motivation for the acquisition of raiding behavior as males grow older. Third, increased age may directly raise the probability of exposure to crop raiding because older individuals are more likely than younger ones to have had repeated opportunities over their life time to encounter crops or learn crop raiding from others. Fourth, life history theory predicts that when behaviors that enhance current reproductive success are associated with risks to survival, males in their prime should be more likely than others to engage in risky behaviors [23], [24]. Young male elephants are expected to have a low current reproductive potential and high future reproductive potential and should engage in less risky behaviors such as crop raiding than males near their reproductive peak.

Social networks provide a way to measure patterns of exposure of individual animals to conspecifics. The behaviors of individuals in an animal's network can determine its probability of acquiring those behaviors through social learning [25]. Social learning is likely to be better than solitary learning when the cost of exploratory learning is high and when socially acquired information is reliable [26]–[29]. Crop raiding is a high risk behavior because many male elephants are killed or injured as a result of conflict [3], [30] suggesting that individuals who fail to minimize detection by farmers while raiding, risk injury or death from farmers defending their crops. Because raiding is a risky behavior, animals are expected to learn from reliable sources such as experienced or older associates [31], [32], or by observing a behavior performed by several individuals or repeatedly by a familiar individual [33].

The structure of association networks can also influence the transmission and spread of socially learned behavior in populations [34], [35] and may set limits on the number of individuals that will acquire the behavior. Features of network structure such as network density (the number of observed pairwise associations as a fraction of all possible pairwise associations), community structure (the tendency for individuals in a population to form dense association within clusters and weak associations between clusters) and homophily (the tendency of individuals to associate with others with similar attributes like age or risk taking behavior), are known from theoretical models to influence the spread of behavior [36]–[38]. For example the spread of complex or risky behaviors that require social reinforcement or a critical threshold of exposure to the behavior by naïve individuals in the population can be slowed down or even halted if the association network is sparse. On the other hand the presence of community structure or distinct social groups in a population will enhance the spread of behavior within social groups because of the presence of dense associations within clusters whereas the sparse associations between clusters in populations with a community structure will limit the spread of behavior across social groups. Associations among individuals with a similar propensity to take risks may facilitate the spread of risky behavior within a social group but may hinder the spread of behavior between groups with different propensities for risk-taking.

Studies of male elephant association network properties such as the density of pairwise associations, community structure and association based on age have received little attention [39] and yet they are likely to influence whether social learning or life history factors drive the prevalence of crop raiding behavior in male elephants. For example if males strongly associate with age peers, then life history factors will be a major force driving the acquisition of raiding behavior more than social learning. On the other hand if age is correlated with raiding behavior and if most young raiders associate with older raiders, then social learning will dominate life history as a driver of raiding behavior in a population.

In this paper, we tested several predictions. First we tested the prediction that the probability of being a raider increased with age. Second we tested the prediction that the probability of being a raider was higher for males whose top associates were raiders than for males whose top associates were non-raiders. Third, we tested the prediction that the probability of a male being a raider rises with increase in the relative age of his associates who are raiders. Fourth, we examined elephant social network properties: density of associations, community structure, and association based on age similarity for their potential to influence the spread of raiding behavior in the population. After establishing the presence of community structure or social groups, we tested the prediction that (a) raiders are distributed non-randomly across elephant social groups, (b) the mean age of individuals in each social group is not different from a random age sample taken from the population.

## Materials and Methods

### Ethics Statement

The protocols used in this study were approved by the Institutional Animal Care & Use Committee (IACUC), Duke University and Medical Center under Registry Number: A333-05-12. In Kenya, permission to conduct this research in the Amboseli National Park and surrounding areas was approved by the Office of the President of the Government of Kenya through permit number MOEST 13/001/35C 225.

### Study Area

This study was conducted in the Amboseli National park and adjacent areas, which constitute part of the 8,000 km^2^ of the larger Amboseli ecosystem located in southern Kenya and at the northern slopes of Mt. Kilimanjaro. Rainfall in the ecosystem varies spatially and temporally with an average annual rainfall of 340 mm recorded within the Amboseli National Park [40]. Rainfall occurs during the long rainy season (March to May) and the short rainy season (November to December). The vegetation also varies spatially. The dominant vegetation is open or bushed grassland in the northern and eastern areas of the ecosystem, and Acacia-dominated grasslands in the south. Interspersing these vegetation types are swamps and swamp vegetation.

Human agriculture and settlements occur 10 km to the east in Namelok, and about 20 km to the east and south east of Amboseli National Park in the Kimana and Loitokitok farming areas [40]. The main crops grown include maize, onions, tomatoes and beans. All these crops are raided by elephants. We monitored crop raiding in Namelok, Isinet in the Kimana farming area, and Sompet in the Loitokitok farming area [9].

### Study Population

This study focused on the Amboseli elephant population, currently consisting of about ∼1400 elephants. Of these, ∼365 males and ∼510 females were 10 years or older by August 2007. This population has been intensively studied since 1972 by the Amboseli Elephant Research Project (AERP). All elephants in the Amboseli population are individually known and are identified using natural tears, notches, holes and vein patterns on ear pinnae [41]. Elephants are also identified from tusk characteristics (size, shape and configuration, one-tusked, broken or intact), and natural body marks [41]. We used photographic identities, maintained by AERP, on all Amboseli males and identities compiled by the first author to confirm individual identities in the field. This population is free ranging and uses an area of nearly 8000 km^2^, including Amboseli National Park and surrounding Maasai ranches in Southern Kenya [42]. The range of the Amboseli elephant population overlaps with the range used by elephant populations from Tsavo and Chyulu in the east and those of Kilimanjaro in the south [42]. All known Amboseli elephants have ages assigned to them; elephants born since 1975 have their ages estimated to within 2 weeks, those born between 1972 and 1974 have ages estimated to within a few months, elephants born between 1969 and 1971 have ages estimated to within 1 year, and elephants born before 1969 have ages estimated to within 2–5 years. The ages for animals born since 1975 are based on the time difference between when a mother was last seen without a calf and when she was first seen with a newborn calf (usually a several-week period at the most). All age estimations are validated from long-term observations of growth and body shape, as well as from ages based on tooth wear and replacement when dead [30].

### Estimation of Male Associations

We collected association data during sightings of all-male elephant groups from June to December of 2005 to 2007; these observations were carried out opportunistically because locating elephants was not predictable. We searched for male elephants daily by driving to areas where elephants were likely to be sighted. When we sighted elephants in all-male groups, we recorded the identities of individuals in the group. We defined an elephant group as a spatially cohesive and behaviourally coordinated aggregation of two or more elephants. An elephant group was defined as spatially cohesive if individuals were aggregated within a radius of 100 m and if they were orientated or moving in the same direction. Elephants were considered to be behaviourally coordinated if they had similar activity patterns or interacted during a 10–30 min observation window.

After data collection was complete, we strove to obtain a realistic and an unbiased representation of male association patterns by choosing individuals for whom we had a minimum of 15 sightings when they were in all-male groups during the study period. This produced a sample of 58 individuals (15% of the male population 10 years and older) defined by the following measures: mean of 36 sightings, median of 18 sightings, mode of 46 sightings and a maximum of 107 sightings. We also chose individuals whose frequent associates were sighted at least 15 times, to eliminate individuals from the sample whose major associates were not sampled intensively. From these data we estimated the association of dyads using a simple association ratio or Association Index (*AI*), where *AI* = *N_AB_*/(*N_A_*+*N_B_*+*N_AB_*). *N_AB_* is the number of times individual *A* and *B* are sighted in the same group, and *N_A_* and *N_B_* are the number of times individuals *A* and *B* are sighted in different groups. We then rank-ordered each male's associates from 1 to 57, with 1 being the male he most frequently associated with (i.e. his closest associate) and 57 being the male he least frequently associated with or did not associate with.

### Identification of Crop-Raiders

**Figure 1:**
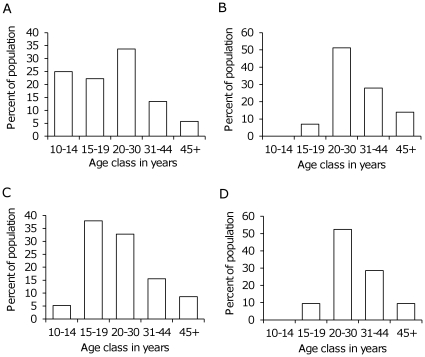
The age distribution of males in the Amboseli population and in our sample. A: The age distribution of all male elephants in the Amboseli population that were 10 years and older (n = 365). B: The age distribution of all the male crop raiders that were detected from the Amboseli population (n = 43). C: The age distribution of all elephants in our study sample (n = 58 males). D: The age distribution of crop raiders in our study sample (n = 21). The Age distribution of our sample of 58 individuals (C) was not significantly different from the age distribution of the entire Amboseli population of 365 individuals (A) (Kolmogorov-Smirnov test, D = 0.800, P = 0.079). Similarly, the age distribution of raiders in our study sample (D) was not different from that for all the raiders detected in the Amboseli elephant population (B) (Kolmogorov-Smirnov test, n = 43, D = 0.4, P = 0.810).

Data on crop raiding was collected independently of the association data using a different sampling protocol. We identified 43 individual crop raiders from direct observations and from genetic analysis of feces collected from raided farmland over a three year period starting in May 2005 and ending in November 2007. We followed elephant tracks from raided fields during the day until we located and identified all individual raiders. When we were not able to locate raiders, we collected elephant fecal samples from raided crop fields and used molecular-genetic techniques to identify individuals [9]. Although we detected 43 distinct raiding individuals, we estimated that there were possibly 40 additional elephants that we could not detect given our sampling intensity and the patterns of raiding by elephants [9]. In order to minimize the risk of assigning raiders to a non-raiding category, we restricted our sample in the current analysis to 58 elephants comprising 21 raiders and 37 non-raiders that we frequently observed during association studies. These 37 non-raiders were chosen because the frequency with which we observed them during our behavioral study made us relatively confident that we had no undetected raiders in this group. We compared the age distribution of this sample of 21 raiders and 37 non-raiders with the age distribution of male elephants older than 10 years in the population (Figure 1) to examine any age bias in our sample. We found that the age distribution of our sample was not significantly different from that of the entire male Amboseli population (Figure 1).

In all the statistical analyses that follow, subjects were designated as raiders (represented by the numeral 1) or non-raiders (represented by zero). The relative age of an associate was defined as the age of a focal male subtracted from the age of his associate. Values of relative age or age difference were negative for associates who were younger than the focal male and positive for associates who were older than the focal male. All probability values reported in the results are for two tailed statistical tests and significance was assessed at P>0.05.

### Predicting Crop Raiding Behavior in Males from Age and Association data

To predict the crop raiding status of a male from his age and association data, we employed logistic regression models. In a logistic model, the probability of being a raider (*P*) is estimated using the formula: *P = exp (βo+∑βiXi)/1+exp (βo+∑ βiXi)*, where *exp* is the exponent, *βo* is the intercept, and *βi* is vector of coefficients corresponding to *Xi* predictor variables: age of the focal male, raiding status of associate and relative age of associate. To estimate these coefficients, we maximized the likelihood function for a vector of parameter *β* using the Newton-Raphson algorithm [43]. For a model with the intercept and age of a focal male as the only predictors or raiding probability, we evaluated the probability that the estimated coefficients were different from zero using the Wald statistic [44]. However, for models in which we used the characteristics of associates as predictors, we evaluated how different our logistic coefficients were from a random expectation using Monte Carlo analyses (Randomization tests) because associations of an individual may not be independent of the associations of his associates. Such independence in data can inflate degrees of freedom, producing biased probabilities that coefficients are different from zero [45]. For each hypothesis, we shuffled predictor variables against the raiding status of focal males and obtained 1000 random datasets. We then performed logistic regression analyses on these data and extracted the coefficient values of predictor variables. Coefficients from random data represented a distribution of expected values for the null hypothesis that the raiding status of a male is not influenced by the raiding status or traits of his associates. We determined the proportion of absolute coefficient values from random data that were equal to or more extreme than the absolute coefficients determined from the original data. We used this proportion as the probability that observed values were different from a random expectation. Monte Carlo analyses were conducted using PopTools version 3.2.3 [46] and XLSTAT version 2010.4.01 (Addinsoft, New York).

To test the prediction that the probability of being a raider increased with an increase in age, we performed logistic regression analyses using data for the entire male population of 365 Amboseli elephants that were 10 years and older. In addition, we also performed a similar analysis on the subset of only 58 males for whom we had association data for and who thus formed the focus of the rest of the analyses. For the larger dataset, we grouped elephants into age classes corresponding to major life history milestones and then performed a logistic regression on the raiding status of elephants as a dependent variable and their age classes as an independent categorical variable. We grouped males into five age categories. Age classes 10–14 years, and 15–19 years correspond to early and late dispersal phases respectively [39], age class 20–30 years corresponds to the initiation of first musth, age class 31–44 corresponds to a period of rapid ascent in reproductive potential and ages 45+ years corresponds to the age of attainment of a peak in reproduction [19], [20]. We ran this model to specifically test whether males in their reproductive prime were more likely to be raiders compared to all other male classes.

To test whether the raiding status of a male's top five associates influenced his probability of being a raider we first ranked each male's associates from most close associate (1) to least close associate (57) for associates with the largest and the smallest AI values respectively. We then performed single variable logistic regression analyses with the raiding status of the focal male as a dependent variable and the raiding status for each of the five closest associates as independent variable. We evaluated the significance of our logistic coefficients for each of the five closest associates using randomization tests. We generated 1000 datasets. In each dataset, each male retained their raiding status but their top associates raiding status was randomly assigned a raiding status from one of his 57 potential associates. We performed logistic regression analyses on these randomized datasets and extracted the coefficients for the intercept and raiding status of the randomly chosen closest associate. We used the coefficients from the randomized data to evaluate the hypothesis that a male's probability of being a raider was not predicted by the raiding status of their associate regardless of the associate's rank.

We next used logistic regression to test whether a male's probability of being a raider was predicted by the number of his five closest associates who were raiders. We compared this model with one that included the raiding status of closest and second closest associates as two separate independent variables in a multiple logistic regression framework using Akaike's Information Criterion (AIC). The model with the lowest AIC value was selected as the most likely model. We evaluated the statistical significance of the observed logistic coefficients of the selected model by comparing them with the null distribution of logistic coefficients obtained from analyses of random data generated by shuffling covariates from the original data.

To test the prediction that males were more likely to be raiders if their associates were older raiders than if their associates where younger raiders, we performed a logistic regression analysis on a males raiding status as a dependent variable, and (1) a males own age in years (2) the raiding status of his first closest associate (3) the raiding status of his second closest associates, and (4) the relative age of the top associate (i.e. age of associate minus the age of focal male) (5) the relative age of the second associate (6) the interaction between an associates relative age and raiding status of his top associate and (7) the interaction between an associates relative age and raiding status of his second associate as independent variables. We evaluated the significance of the logistic coefficients we obtained using a null distribution of coefficient values generated by performing logistic regression analyses on 1000 randomized data sets. We generated each of these random datasets by randomly assigning a raiding status and relative age to the associates of each focal individual from his pool of 57 potential associates.

We predicted a positive coefficient of the interaction between an associate's raiding status and relative age on the focal male's raiding status. A positive interaction means that the probability of a focal male being a raider increases with the increase in relative age of his associates who are raiders more than with an increase in relative age of associates who are not raiders. A statistically significant difference in slopes of raiding probability suggests that younger individuals are more likely to be learning from older individuals rather than older males learning from younger males.

### Analysis of the Structure of the Male Elephant Social Network

We employed exponential random graph (ERG) analyses to examine the density of male elephant associations, the strength of clustering in these associations and to determine if males associated based on age similarity. We also performed ERG to confirm that males with similar raiding status were more associated than males with dissimilar raiding status as predicted by randomization tests. ERG analyses were performed on a directed binary elephant association network generated by connecting individuals (or nodes) with closest associates whose attributes we found to predict his raiding behaviour using randomization tests. An ERG model is used to express the probability (*X*) of observing a network (*x*) on a fixed set of nodes (*N*) as a function of certain network configurations or subgraphs. These network configurations, also called dependency structures or endogenous effects, are represented as parameters (*θ*) in the ERG model. The ERG model is expressed as Pr(*X* = *x*) = (1/κ) exp (∑*_ij_θ_ij_z_ij_*(*x*)), where *θ_ij_* represents parameters *i* to *j*, *z_ij_* (*x*) represents counts of configurations corresponding to model parameters *i* to *j* in the observed network (*x*), and κ is a normalizing constant [47]. Individual male (node) attributes are incorporated into the ERG model as covariate parameters (*y*) so that the probability *x* is conditional on covariates *y* [48].

For the ERG analyses, we incorporated parameters for structural configurations (endogenous effects) known to capture salient features of social networks such as association density (relationship between the number of observed associations and the possible number of associations among individuals in the network), reciprocity (the tendency for mutual associations to occur in the network such that if A is top associate of B, then B is also top associate of A), the distribution of the number of associates per individual (or degree distribution) and transitivity (the tendency for associates of two associated individuals to also be associated) [49]. We also included parameters for source nodes, some complex star configurations (e.g. AinS, Ain1out-star, 1inAout-star and AinAout-star, see Figure S1) and one transitivity configuration (e.g. AT-T, see Figure S1). Star-based parameters and parameters for source nodes model the distribution of associations across individuals [49]. The transitivity parameters model clustering in a social network, and when combined with Markov configurations, they capture complex dependence and enable parameters to converge during model fitting [32]. The reciprocity parameter was included because reciprocity is prevalent in social networks. Additionally, we included parameters for attributes of individual male elephants such as age and raiding status in order to test the hypothesis that elephants associate on the basis of age proximity and raiding status.

We fitted ERG model with above parameters to the elephant association network using the Markov Chain Monte Carlo Maximum Likelihood Estimation procedure employing the Robbins–Monro algorithm [50]. This procedure generates networks from an initial guess at parameters estimates and then iteratively updates these initial parameters at each simulation step to obtain refined parameters that closely replicate the observed network. Final parameter values obtained were evaluated for convergence using the *t* ratio: the difference between the value of a parameter determined from the observed network and the mean of the parameter determined from the sample of simulated networks. An absolute *t* ratio less than 0.1 for all parameters used in model fitting and an absolute *t* ratio of less than 1 for the parameters not used in model-fitting were used to indicate model convergence. The probability values for the fitted parameters were calculated from the variance of parameters obtained from 1000 simulated networks. All ERG analyses were performed using PNet [51].

When we obtained zero as an estimate of a fitted parameter, we interpreted that as an indication that the effect being modelled occurred by chance. We interpreted a positive parameter as an indication that the effect modelled was more prevalent and occurred more often than expected by chance. A negative parameter, on the other hand, indicated that the effect was less prevalent than expected by chance alone. However, for the age covariate, a significant negative parameter estimate indicated a tendency for associated elephants to be closer in age more than predicted by chance because we used absolute age differences to fit our model.

To visualize the elephant association network and to identify social clusters in the elephant network, we used the Girvan-Newman modularity maximization algorithm implemented in NetDraw [52]. The Girvan-Newman algorithm, finds an objective method for dividing the network into clusters of individuals with who are more associated with each other and less associated with individuals outside their cluster. This algorithm maximises the modularity quotient Q as a way to objectively determine the number of clusters in a network. The strength of clustering ranges anywhere between zero and one, with zero indicating no clustering and a one indicating the population consist of discrete clusters. We estimated the probability that our observed Q was significantly different from a random expectation by calculating the proportion of Q values estimated from randomly generated networks that are equal to or more extreme than Q estimated from the observed network. We generated random networks by randomly shuffling edges (associations) between nodes (individual elephants) in our network using NetDraw.

After establishing that the modularity of the elephant association network was significantly different from a random expectation, we tested whether the proportion of raiders and the mean age of males in each cluster were significantly different from a random sample from the population. We determined the expected proportion of raiders and mean age of males in each cluster by randomly shuffling the raiding status and ages of individual elephants across clusters while holding the number of individuals per cluster constant. We created 1000 randomized datasets and for each data set, we estimated the proportion of raiders and the mean age of individuals in each clusters. We calculated the proportion of raiders in each cluster and the mean age of individuals in each cluster from 1000 randomized datasets that were equal to or more extreme than the proportion of raiders and the average age in each cluster estimated from the original data. We used this ratio as an estimate of the probability that our observed proportion or mean age in years in each cluster was significantly different from a random expectation.

## Results

### Age Predicted the Probability of being a Crop-Raider

The probability of being a crop raider was positively predicted by a males' own age (intercept = −3.925, P<0.001; age = 0.0730, P<0.001, n = 365). This pattern did not change when we used only the subset of the dataset from the Amboseli population for which we had adequate association data and which formed the focus for the rest of the analyses (intercept = −2.468, P = 0.002; age = 0.0740, P = 0.010, n = 58). When we used age class as an independent categorical variable with age classes corresponding with major life history milestones, we detected a dramatic increase in the odds of being a raider for males initiating reproduction and a near doubling of these odds when males attain their reproductive prime age or 45+ years (Table 1).

**Table 1:** Logistic regression coefficients showing that age class categories in years predicted the probability that a male was a raider.

Independent variable	Number of males (Raiders)	Coefficient ± Standard error	Chi-Square	Odds ratio	Probability value
**Intercept**		−5.209±1.426	13.349		0.000
**10–14** 1	91 (0)	0.000±0.000			
**15–19**	81 (3)	2.099±1.528	1.887	8.159	0.170
**20–30**	123 (22)	3.703±1.445	6.568	40.566	0.010
**31–44**	49 (12)	4.111±1.464	7.890	61.000	0.005
**45+**	21 (6)	4.340±1.504	8.329	76.742	0.004

The probability values show whether the coefficients were significantly different from zero for each age class.

1 Age class 10–14 years was used as a baseline age class and as result its' coefficient is set to zero. We used data for the all males over 10 years of age from Amboseli National Park (n = 365 elephants). The total number of males in each age class is indicated and the number of crop raiders in each age class is shown in parenthesis. The odds ratio is the exponent of the coefficient and provides a measure of how more likely a male from a named age class is to be a raider compared to the raiding status of the baseline age class.

### A Male's Raiding Status was Predicted by the Raiding Status of his Close Associates

The raiding status of top associates was a strong predictor of a male's raiding status. Specifically, the probability of being a raider was higher than predicted by chance for a male whose closest associate was a raider (intercept = −1.19, P = 0.017; raiding status of first associate = 2.379, P = 0.001, n = 58 elephants) or whose second closest associate was a raider (intercept = −1.513, P = 0.030; raiding status of second associate = 2.113, P = 0.003, n = 58 elephants). However, a male's raiding status was not predicted by the raiding status of his third (intercept = −0.389, P = 0.670; raiding status of third associate = 0, P = 1.000, n = 58 elephants), fourth (intercept = −0.188, P = 0.910; raiding status of fourth associate = 0, P = 1.000, n = 58 elephants), or fifth closest associate (intercept = −0.747, P = 0.151; Raiding status of fifth associate = 0.636, P = 0.195, n = 58 elephants). Although a male's raiding status was predicted by the number of his five top associates who were crop raiders (intercept = −1.889, P = 0.001; proportion of top five associates who were raiders = 0.650, P = 0.021, n = 58 elephants), the model that included the raiding status of a males' top two associates as two separate independent variables (Table 2) had considerable support (Δ AIC = 8.704). Exponential random graph analyses based on the two closest associates also confirmed that there was a strong pairwise association of elephants based on similarity in raiding status (Table 3).

**Table 2:** Logistic regression coefficients for predictors of a male's raiding status showing the probability that the observed coefficient values were significantly different from a random expectation (n = 58 elephants).

Independent variables	Observed coefficient	Mean±Standard error of expected coefficient	Probability value
**Intercept**	−1.740	−0.454±0.012	0.002
**Raiding status of closest associate**	1.783	−0.102±0.014	0.005
**Raiding status of second closest associate**	1.596	−0.133±0.016	0.010

**Table 3:** Exponential random graph coefficients of an elephant association network showing that the elephant network had a sparse density of associations, a strong clustering, a strong association by raiding status and a weak association by age (n = 58 elephants).

Independent variables	Estimated value	Standard error	t-ratio	Probability value
**Density of associations**	−5.297	1.343	0.025	0.000
**Transitivity (AT-T)** 1	0.606	0.105	0.068	0.000
**Reciprocal associations**	4.781	0.564	−0.029	0.000
**AinAoutS (Alt-in-alt-out-star)**	6.357	1.322	−0.049	0.000
**1inAoutS (1-in-alt-out-star)**	1.745	0.681	0.034	0.011
**Ain1outS (Alt-in-1-out-Star)**	−5.243	0.722	−0.044	0.000
**Source node**	1.678	1.037	0.076	0.106
**AinS (Alt-in-Star)**	0.241	0.971	0.054	0.804
**Association by raiding status**	0.672	0.319	0.019	0.035
**Association by age** 2	−0.027	0.010	−0.024	0.007
**Reciprocity of associations based on raiding status**	−0.251	0.670	−0.022	0.708

1 Transitivity parameter indicates clustering and a schematic representation of this and other parameters in this table are shown in Figure S1.

2 Data on age differences between dyads was used to test whether associations in the network are based on age proximity in this analysis.

### A Male's Probability of being a Raider was Higher when his Associates were Older Raiders than when his Associates were Younger Raiders

A model predicting the probability of a male's raiding status from his age, the raiding status and relative ages of his two closest associates and the interaction between the raiding status and relative ages of his closest associates had the lowest AIC compared to a model with raiding statuses of the two closest associates only (Δ AIC = 15.312) or a model with age of focal male and the raiding status of his associates (Δ AIC = 14.923).

In the model with the lowest AIC, we found a strong positive interaction coefficient between the raiding status and relative age of his second closest associate but not his first closest associate (Table 4). This positive interaction indicates that the probability of a male being a raider increased more with the relative age of his second closest associates who were raiders than with relative age of his second closest associates who were not raiders. This difference in slopes of raiding probability suggests that younger males were more likely learning from older individuals rather than older males learning from younger individuals (Table 4). We were unable to detect an interaction between a top closest associate's relative age and raiding status on a male's raiding status, perhaps because most raiders whose closest associates were raiders were slightly older and had closest associates with a similar age to their own (Mean age of focal males was 33.1years and mean age of closest associates was 30 years; t test; d.f. = 10; P = 0.54). On the other hand, males who were raiders and whose second closest associates were also raiders had significantly older associates (Mean age of focal males was 30.6 years and mean age of second closest associates was 37. 1 years; P = 0.05; t test; d.f. = 14). Results from a joint model, i.e. a model with the lowest AIC, also confirmed our earlier results presented in Table 1 showing that older males were more likely to be raiders than younger males and results in Table 2 showing that a male elephant was more likely to be a raider if his closest associates were raiders.

**Table 4:** Logistic regression coefficients of independent variables, showing the probability that the observed values were significantly different from a random expectation (n = 58 elephants).

Independent variables	Observed coefficient	Mean ± Standard Error of expected coefficient	Probability value
**Intercept**	−5.232	−0.506±0.029	0.001
**Age of focal male**	0.141	−0.001±0.001	0.002
**Relative age of closest associate**	0.053	−0.031±0.002	0.224
**Relative age of second closest associate**	0.013	−0.027±0.001	0.267
**Raiding status of closest associate**	2.550	−0.104±0.018	0.004
**Raiding status of second closest associate**	0.033	−0.117±0.017	0.379
**Interaction between raiding status and relative age of top associate**	−0.029	0.000±0.002	0.367
**Interaction between raiding status and relative age of second associate**	0.188	−0.003±0.001	0.009

### Metrics of Elephant Social Networks

**Figure 2:**
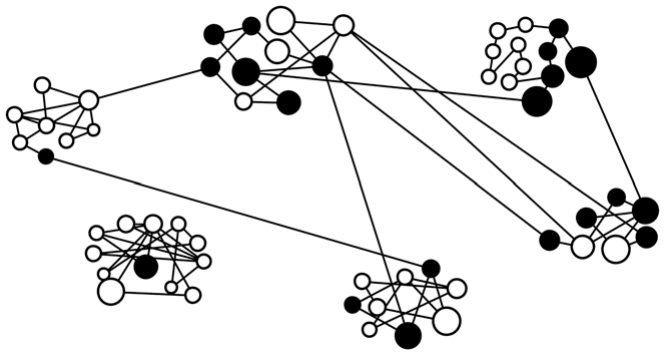
An association network of male elephants showing a strong community structure (Modularity, Q = 0.729). The nodes represent 58 individual male elephants and the size of the node is proportional to age of an individual male. Black circles (nodes) indicate raiders and the white circles indicate non-raiders. Nodes are grouped into six clusters using the Girvan-Newman algorithm in NetDraw. Clusters in the top row from left, center and right are identified as A, B and C respectively and clusters in the bottom row are identified as D, E, F from left, center and right respectively.

The density of association in the elephant social network was significantly sparse (Table 3). The elephant association network had a significant amount of clustering assessed using the transitivity parameter in the ERG analyses (Table 3) and a strong community structure assessed using Girvan-Newman modularity analysis (Figure 2). The modularity for the observed network (Q = 0.729) was significantly different from a random expectation (mean Q ± standard deviation = 0.250±0.044, P = 0.001).

At the cluster level, the distribution of individuals by raiding status and by age across association clusters was as expected by chance in five of six clusters constituting our network (Table 5 & Table 6). Only one cluster had a significantly smaller proportion of raiders than expected by chance (Table 5) and another cluster had significantly younger elephants than expected by chance (Table 6).

**Table 5:** The observed and expected proportion of raiders in six clusters (A to F) showing the probability that the observed proportion of raiders in each cluster was significantly different from the expected mean proportion.

Cluster identity^1^	Cluster size	Observed proportion of raiders	Mean±Standard error of the expected proportion of raiders	Probability value
**A**	8	0.125	0.359±0.005	0.230
**B**	10	0.600	0.358±0.004	0.152
**C**	12	0.417	0.369±0.004	0.964
**D**	12	0.083	0.360±0.004	0.038
**E**	9	0.333	0.370±0.005	0.906
**F**	7	0.714	0.352±0.005	0.076

1 Corresponding cluster identities are shown as a diagram of a network of association clusters in Figure 2.

**Table 6:** The observed and expected mean age and standard error for six clusters (A–F) showing the probability that the observed mean age values were not significantly different from the expected mean age values for each cluster.

Cluster identity^1^	Cluster size	Mean ± Standard error of observed age in years	Mean ± Standard error of expected age in years	Probability value
**A**	8	17.875±1.529	25.201±0.106	0.022
**B**	10	31.500±2.861	25.206±0.099	0.064
**C**	12	24.833±3.649	25.202±0.083	0.902
**D**	12	20.583±2.624	25.211±0.085	0.078
**E**	9	25.667±3.444	25.149±0.100	0.846
**F**	7	32.429±3.213	25.149±0.116	0.060

1 Cluster identities shown here correspond to identities shown in Figure 2.

## Discussion

Our results show that the probability of crop-raiding by male elephants increased as a function of age such that the odds of being a crop raider rose dramatically at the age at which reproduction is initiated and the odds nearly doubled for males at their reproductive peak. This result strongly suggests that crop raiding is linked to the increasing energetic costs associated with reproduction or increased risk taking behavior associated with attainment of a peak in reproduction at age 45 years in male elephants [19], [20]. Although an increase in exposure to raiding through conspecifics or through trial and error learning is likely as males become older, we would expect a steady and a less dramatic rise in the odds of raiding if exposure was a major driver of raiding behavior in elephants. In many mammals, increase in energy requirements has been demonstrated to be associated with age of reproductive initiation and age of attainment of a peak in reproduction [16]–[18]. These increasing energy needs may provide males with the motivation to initiate crop raiding through trial and error learning or by learning from their associates.

Our results also indicate that raiding by males was predicted by the raiding status of their closest associates and this effect was stronger if their associates were older. These results suggest that raiding is acquired through social learning from older males that are raiders. Our observations support results from experimental studies of social learning in other vertebrates showing that animals show a bias towards learning from individuals that are older, more experienced or familiar. For example in one study, young female guppies learned mate preferences from older females [53]. In another study, nine-spined sticklebacks learned foraging tactics from larger demonstrators more than they learned from younger demonstrators [31]. In mammals, garbage feeding behavior in free ranging bears has been demonstrated to be socially learnt by infants from mothers [54]. Our findings contribute to a growing body of information regarding the role of social learning in the spread of new foraging behaviors in wild vertebrate populations [2], [25], [54]–[56].

Although a shared spatial environment could allow associates to adopt similar foraging behaviors through independent exploratory learning irrespective of social learning, this is a very unlikely explanation for the pattern we observed. Elephants are not territorial, and males particularly range widely and have large home ranges [57]. Areas with crops in Amboseli are within 20 km of Amboseli National Park, well within a day's elephant ranging distance from the core areas used by elephants in this population [58]. The above observations suggest that all male elephants in our population are expected to raid if access to crops was the only factor influencing their probability of crop raiding.

Crop raiding is a high-risk and high-gain foraging strategy: successful raiders in Amboseli [59] and in other elephant populations [60], [61] derive substantial nutritional benefits from crops. At the same time, a large number of elephants are killed or injured annually as a result of crop raiding and other human elephant conflict situations by both farmers and conservation agencies [3], [4]. In the Amboseli elephant population, Moss [30] reported that 65% of adult elephant mortality was caused by humans as a result of conflict. In these circumstances it would be adaptive to learn from reliable individuals with raiding experience and knowledge on how to avoid detection and minimize risk, rather than by trial and error learning. Empirical studies on other vertebrate species have shown that learning from more experienced individuals is adaptive when social learning is associated with substantive benefits, and when errors associated with individual exploratory learning are costly [32].

For crop-raiding elephants, minimizing risk and avoiding detection by farmers may entail raiding late in the night or raiding mainly on moonless nights [62]. During raiding, elephants may reduce individual risks by raiding in large groups. In fact male elephants have been observed to form larger group sizes while raiding and smaller groups while foraging on wild plants [14].

In Kenya, the Kenya Wildlife Service policy is to frighten elephants from farms by shooting into the air, but farmers may spear crop raiding elephants illegally. During this study, we observed some crop-raiders with spear injuries presumably incurred during crop raiding. A study examining stress associated with crop raiding in the Amboseli and Maasai-Mara ecosystems in southern Kenya, found that raiders had higher levels of stress than non raiders [12].

We also observed features of the elephant association network that may limit the spread and hence reduce the prevalence of raiding behavior in the population. First, the male elephant network had a significantly low density of associations indicating that males may have limited exposure to raiding behavior from other males within the network. Secondly the elephant social network had strong clustering and community structure. These features of the social network will hinder the transmission of information between elephant social groups because of sparse associations between individuals in different groups, but will enhance transmission within social groups because of the dense associations between individuals in the same social cluster.

Thirdly, we observed a weak association of males based on age similarity. Because age influenced the motivation to raid, this suggests that some males may have initiated raiding through social facilitation among similarly aged individuals, perhaps driven by energetic demands of reproduction. The association based on age similarity was driven by older male elephants [63] suggesting that for older elephants, life history factors were a dominant component of raiding acquisition. However, younger males associating with older males who were raiders also experienced an increased probability of being raiders, suggesting a strong influence of social learning as a dominant process in the acquisition of raiding behavior in younger males.

Cluster level network analyses revealed that, in five of the six clusters in the network, raiders were distributed randomly across clusters. These results were discordant with the results of pairwise association from Monte Carlo and ERG analyses, which showed that elephants associated with other males who were similar to them in raiding status. The discordance in cluster level and pairwise level analyses suggest that elephant association based on similarity in raiding status was driven by local dyadic effects rather than by network cluster effects. The lack of association based on raiding status at the cluster level and its presence at the dyadic network level could be a result of a low density of associations in the network. If the network is less dense within a cluster, most individuals within a cluster will not be connected to each other and this may limit the spread of raiding information even within a cluster. Individual variation in risk taking can also limit the homogenization of clusters resulting in a random distribution of raiders across clusters. If some individuals are risk averse, they may not adopt raiding as a foraging strategy; this in turn will result in clusters with both raiders (risk takers that have been exposed to raiding) and non raiders (risk-averse individuals who have been exposed or not exposed to raiding or risk-prone individuals that have not been exposed to raiding).

Male social clusters had a heterogeneous age distribution. This heterogeneous age distribution and association based on age similarity from ERG network analysis suggest that most males prefer to associate with age peers and some prefer to associate with individuals younger or older than them. The heterogeneity in age distribution within clusters suggests that social learning and life history are likely to simultaneously influence raiding behavior within clusters.

Our findings have implications for the management of human-elephant conflict particularly for populations in which males cause most of the conflict. First, our results demonstrate how social networks and life history milestones may jointly influence the prevalence of learnt behavior. This in turn suggests that maintaining heterogeneous age structure may be important in promoting adaptive learning and response to changing environments in animal populations. Related to this, our results suggest that aversive conditioning techniques, such as pepper spray canisters [64] should be targeted at both older and younger raiders. Targeting older raiders could minimize the spread of raiding behavior through social learning, while targeting younger raiders may deter them from raiding in later years when they attain their reproductive peak. Killing of older raiders, on the other hand, may reduce current raiding but not future raiding as young raiders attaining a reproductive peak may engage in raiding as a result of energetic demands associated with reproduction. Moreover, killing older raiders may also remove sources of ecological knowledge among male elephants. In general, measures that minimize access and exposure of elephants to crops, such as the use of elephant barriers and deterrents, or land use practices compatible with elephant conservation, will remain the most effective strategies in reducing human-elephant conflict [65].

## Supporting Information

Figure S1 **Schematics showing exponential random graph configurations representing parameters we modeled.** The schematics on the left starting with the top most to the bottom most represent star configurations AinAoutS and AinS and a transitivity configuration AT-T respectively. Star configurations; 1inAoutS, and Ain1outS, and a reciprocity configuration are represented by schematics in the top, middle and bottom of the middle column respectively. From top to bottom in the third column are schematics representing source nodes, age difference, raider interaction status, and raider interaction reciprocity respectively. Parameter values for these configurations are shown in Table 3.
(TIFF)
