# Supplementary figures and images for: The Influence of Life History Milestones and Association Networks on Crop-Raiding Behavior in Male African Elephants
Source: PLoS One. 2012 Feb 8;7(2):e31382. doi: 10.1371/journal.pone.0031382 (PMC3275604; doi:10.1371/journal.pone.0031382)

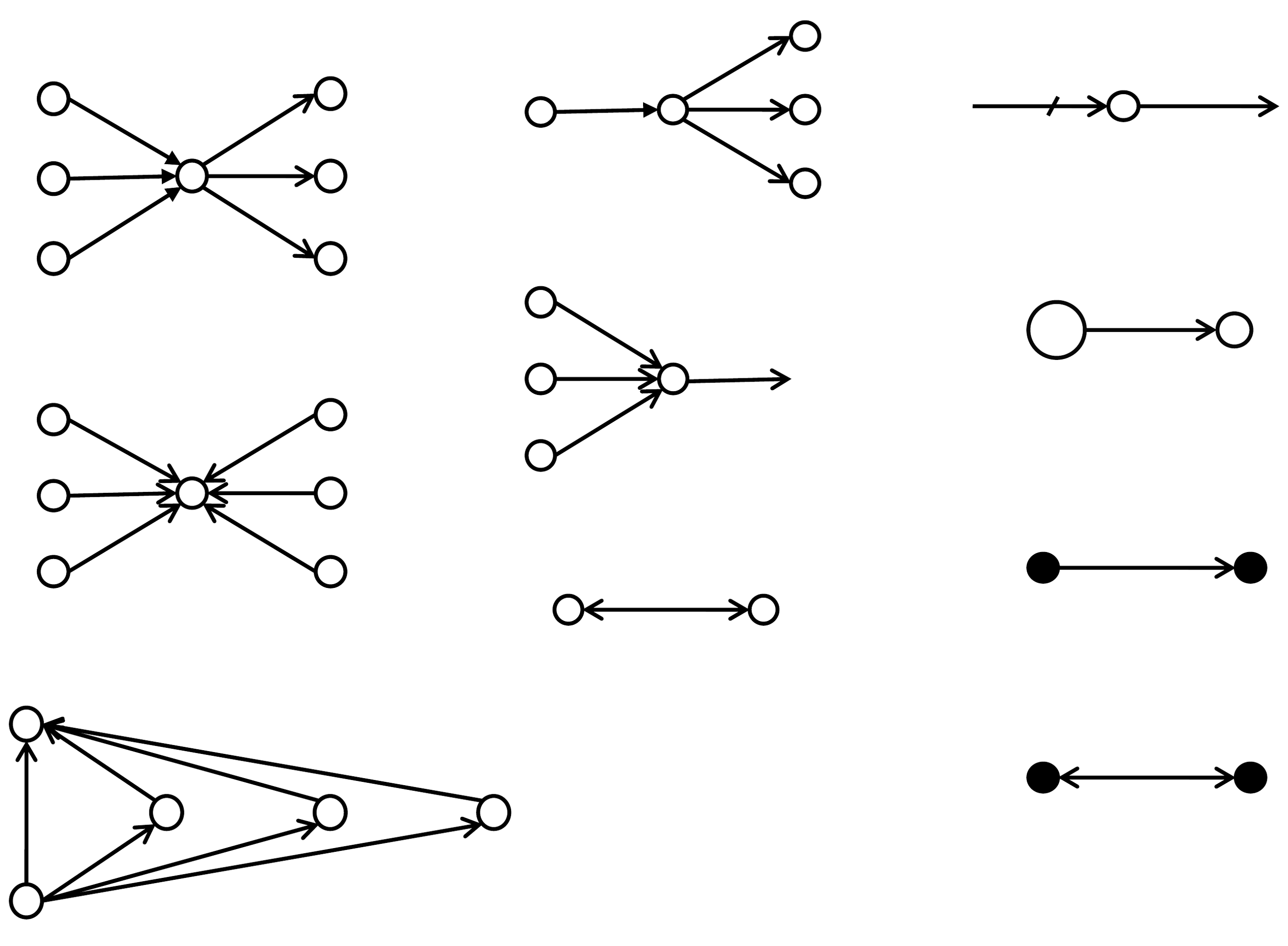

Supplement: Figure S1 — Schematics showing exponential random graph configurations representing parameters we modeled. The schematics on the left starting with the top most to the bottom most represent star configurations AinAoutS and AinS and a transitivity configuration AT-T respectively. Star configurations; 1inAoutS, and Ain1outS, and a reciprocity configuration are represented by schematics in the top, middle and bottom of the middle column respectively. From top to bottom in the third column are schematics representing source nodes, age difference, raider interaction status, and raider interaction reciprocity respectively. Parameter values for these configurations are shown in Table 3. (TIFF) [file pone.0031382.s001.tiff]
